# Urinoma: Prompt Diagnosis and Treatment Can Prevent Abscess Formation, Hydronephrosis, and a Progressive Loss of Renal Function

**DOI:** 10.1155/2018/5456738

**Published:** 2018-09-25

**Authors:** Jason Goldwasser, Razwana Wahdat, James Espinosa, Alan Lucerna

**Affiliations:** ^1^Department of Emergency Medicine, Rowan University SOM/Jefferson, Stratford, NJ, USA; ^2^Department of Emergency Medicine, Virtua Healthcare System, Voorhees, NJ, USA

## Abstract

This case describes a 70-year-old female who presented with right flank pain around the site where a stent had been placed in her right kidney at an outside hospital several months earlier. The patient arrived tachycardic with a leukocytosis and a lactic acidosis. Further imaging revealed a very hydronephrotic right kidney and an extremely large fluid collection in the right retroperitoneum extending into the right flank consistent with leakage of urine from the obstructed right kidney. Prompt treatment of this rare phenomenon is crucial for delay in medical care can lead to abscess, hydronephrosis, electrolyte instability, and a progressive loss of renal function. Treatment for small urinomas is usually conservative as the collection will most often be reabsorbed. Larger urinomas even without systemic signs often necessitate more aggressive medical treatment. A drainage catheter can be placed with ultrasound or CT guidance. Percutaneous nephrostomy tubes are often used as well for additional drainage and decompression. Fluid culture is recommended to guide antibiotic treatment.

## 1. Introduction

Urinary leaks and urinomas are very rare as they result from injury to any part of the urinary collecting system of the GU tract, which in itself is uncommon, making up less than 1% of blunt or penetrating injuries. Urinomas are characterized by urine collections found in the retroperitoneum, most commonly in the perirenal space caused by urinary tract leakage as a direct result of obstruction, trauma, or postinstrumentation. As urine extravasates into the retroperitoneal space, it can cause a local inflammatory response on the surrounding perirenal fat. This leads to lipolysis and an encapsulation of the urine, known as a urinoma.

## 2. Case Presentation

A 70-year-old nonverbal female presented to the emergency department (ED) from home with her daughter who had noted that the patient has been acutely grimacing in pain from even the lightest palpation over her right flank. She had decreased urine output for the prior two days and her family had noticed an enlargement on the right side of her back one day prior to her ED visit. Of note, the patient presented with poor functional status, was totally bedridden, and was on no antibiotics. She also had a renal flow scan several months prior to her presentation, which revealed the absence of blood flow to the left kidney. A nephrectomy was ultimately rejected based on her medical history and current health status. The patient's medical history was significant for CVA with left hemiparesis, sacral stage 4 pressure ulcers, DM, HLD, failure to thrive, and asthma. Her past surgical history included a left double J-stent placement, an appendectomy, and a tracheostomy that was reversed.

**Figure 1:**
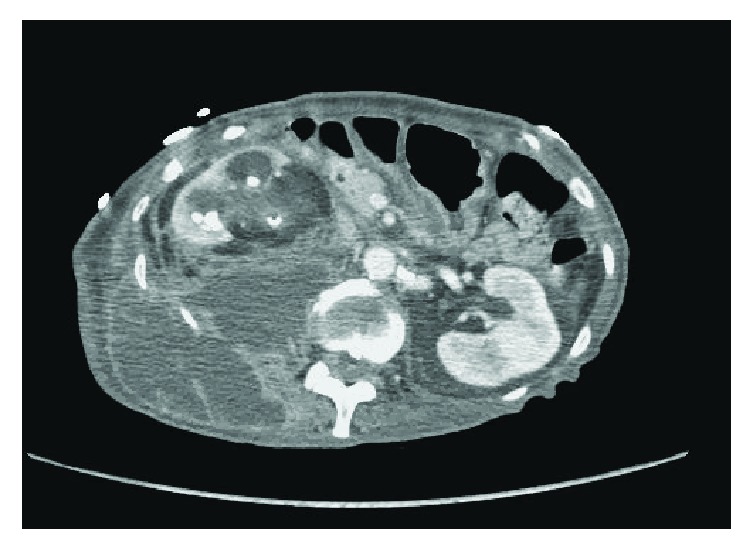
Axial section of a CT scan of the abdomen/pelvis with IV contrast demonstrating severe right hydronephrosis communicating with a large retroperitoneal fluid collection.

**Figure 2:**
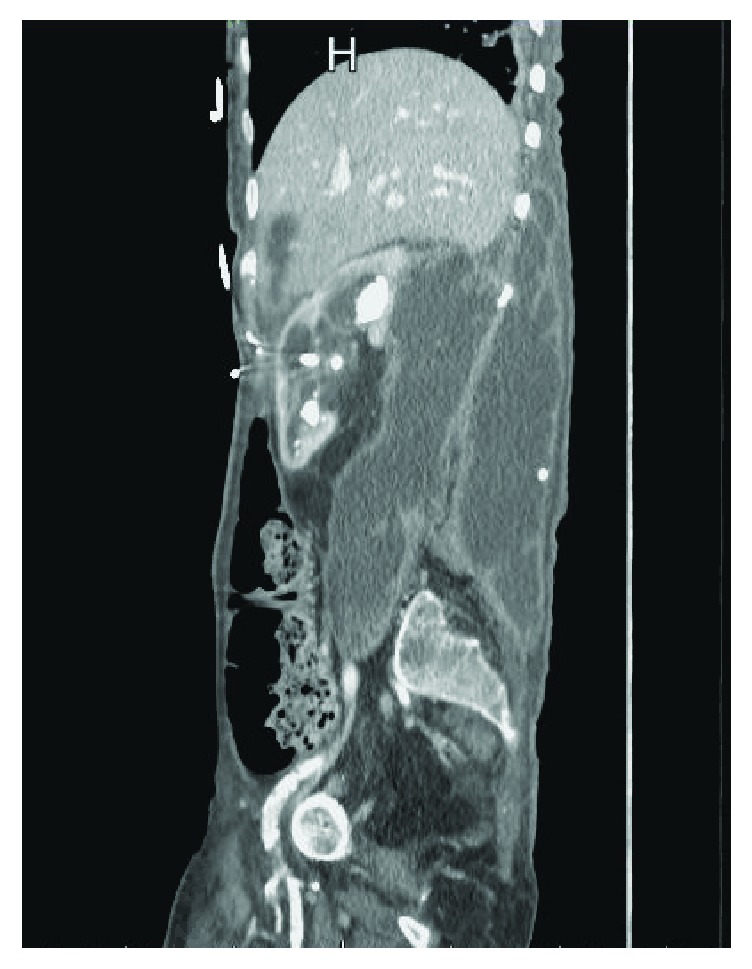
Sagittal section of a CT scan of the abdomen/pelvis with IV contrast revealing severe hydronephrotic right kidney with accompanying urinoma in the retroperitoneum.

**Figure 3:**
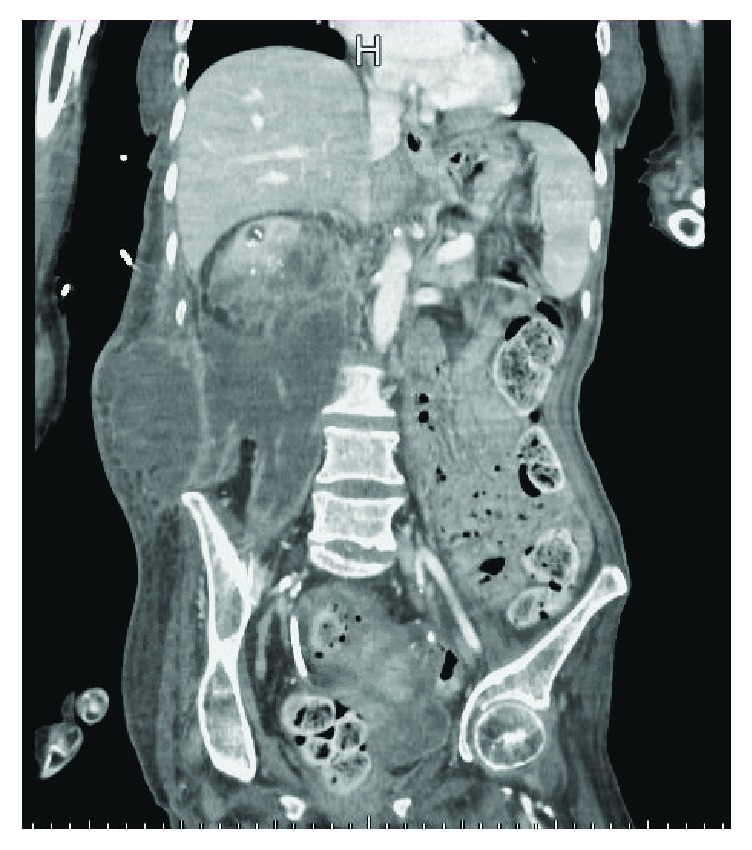
Coronal section of a CT scan if the abdomen/pelvis with IV contrast revealing severe hydronephrotic right kidney with accompany urinoma in the retroperitoneum.

On physical examination, the patient appeared to be in mild distress with a blood pressure of 93/50, heart rate of 116, and otherwise normal vital signs. Abdominal examination was remarkable for tenderness to palpation over the right flank with visible erythematous skin seen in the same area. Results of the initial laboratory tests were significant for a lactate of 2.9, white blood cell count of 18.4, hemoglobin of 7.1, platelet count of 933, albumin of 2.3, and potassium of 5.8, yet with a creatinine of 0.62. She underwent an IV contrast computed tomography (CT) scan of the abdomen and pelvis, which revealed a right hydronephrotic kidney that contained numerous large calcifications (Figures 1 and 2). The right previously placed ureteral stent was in satisfactory position. There was an extremely large fluid collection in the right retroperitoneum extending into the right flank consistent with leakage of urine from the obstructed right kidney (Figure 3).

While, in the ED, urology was consulted and requested interventional radiology to drain the urinoma and to place a Foley catheter to monitor urine output, the attending physician, at the request of interventional radiology, performed a needle aspiration of the loculated fluid collection in the right flank via ultrasound and noted a small amount of purulent and urine-like fluid from two separate areas of collection. The patient was admitted to the intensive care unit for urosepsis, urinoma with abscess, symptomatic anemia, and failure to thrive, while, in the intensive care unit, the patient was evaluated by urology, infectious disease, gastroenterology, wound care, and palliative care.

Urology decided against emergent interventional radiology (IR) drainage as IR did not believe there was an acute need for drainage. It was postulated that the collection was more consistent with a urinoma without an apparent abscess thus not requiring emergent drainage. The patient also had been afebrile and it was reported that there were no fevers noted at home. It was discussed that emergent drainage would be performed if the patient acutely decompensated or became febrile. Additionally, the family refused surgical drainage because of her comorbid conditions. The patient was placed on empiric vancomycin and piperacillin/tazobactam.

The following day interventional radiology placed an 8 French pigtail drainage catheter and drained 610 ml of fluid. A subsequent IV contrast CT scan of the abdomen and pelvis showed the right retroperitoneal collection to be significantly decreased in size and nearly resolved. The contiguous component in the right flank soft tissues was slightly decreased with the drainage catheter in place. The patient was eventually discharged with the drain in place to hospice care.

## 3. Discussion

Urinoma is a rare and unique condition that refers to extravasation of urine from a disruption of the urinary collecting system at any level from the calix to the urethra [1, 2]. It is rare in that ureteral injury comprises less than one percent of blunt or penetrating genitourinary trauma. It is slightly on the rise, however, with the increasing usage of interventional radiological procedures involving the renal system [3]. Clinically, patients can have symptoms ranging from being completely asymptomatic to an acute abdomen, with vague malaise and pain being the most common. Hematuria and urine output changes can be a clue that injury has occurred. Depending on the severity of the injury and the compressive forces, electrolyte imbalances and progressive increases in serum creatinine are laboratory values that can guide the treatment and how emergently intervention needs to be performed [4].

Urinomas are often small at presentation and will reabsorb without intervention in most instances. In cases of a more significant injury, fever, urosepsis, or a larger urinoma that can be expanding and compressing or failing to reabsorb, a procedure is often necessitated by urology or interventional radiology to relieve the urinoma. Failure to do so can quickly lead to abscess, electrolyte imbalance, hydronephrosis, and urosepsis. First line treatment usually involves a drainage catheter placed into the urinoma, in addition to empiric antibiotics with the proper clinical signs and symptoms. When the catheter fails to drain the urinoma appropriately, a percutaneous nephrostomy tube may be placed to facilitate drainage often with a ureteral stent to promote healing [5, 6]. Surgical reconstruction is reserved for the most severe cases. As in most cases, early awareness and prompt treatment are the basis for avoidance of more aggressive measures.

## 4. Conclusion

While urinomas remain a rare entity, one must have a high degree of suspicion for one involved in blunt or penetrating trauma. A dedicated CT scan for a urinoma, which omits the parenchymal phase and involves a low dose noncontrast phase followed by a delayed image phase ten minutes after IV contrast administration, can help with the diagnosis as well as aspiration [7, 8]. A rising serum creatinine can guide the physician as well. Conservative treatment versus various drainage methods might be necessitated to prevent further complications.
